# The impact of obesity surgery on newborn anthropometrics in women with and without polycystic ovary syndrome

**DOI:** 10.1007/s00404-024-07593-0

**Published:** 2024-07-01

**Authors:** Camilla Johannesen Huke, Therese Romsaas, Eszter Vanky, Karoline Huse, Rønnaug Ødegård, Siren Nymo, Dag Arne Lihaug Hoff, Jorunn Sandvik

**Affiliations:** 1 Department of Clinical and Molecular Medicine, Norwegian University of Science and Technology (NTNU), Trondheim, Norway; 2 Department of Obstetrics and Gynecology, St. Olav’s University Hospital, Trondheim University Hospital, Trondheim, Norway; 3 Clinic of Obstetrics and Gynecology, Møre Og Romsdal Hospital Trust, Ålesund, Norway; 4 Centre for Obesity Research, Clinic of Surgery, St. Olav’s University Hospital, Trondheim, Norway; 5 Clinic of Surgery, Nord-Trøndelag Hospital Trust, Namsos, Norway; 6 Departments of Clinical Studies, Møre Og Romsdal Hospital Trust, Ålesund, Norway; 7 Clinic of Surgery, Møre Og Romsdal Hospital Trust, Ålesund, Norway

**Keywords:** PCOS, Bariatric surgery, Post-bariatric pregnancy, Offspring anthropometrics, Obesity surgery

## Abstract

**Purpose:**

Obesity surgery and polycystic ovary syndrome (PCOS) are both associated with increased risk of intrauterine growth restriction. We investigated whether offspring of mothers with PCOS who underwent obesity surgery had an increased risk of deviating birth anthropometrics compared to offspring of mothers without PCOS.

**Methods:**

In this observational study, data from two study databases (BAROBS and PregMet2) were supplemented with data from patient’s records from secondary and tertiary hospitals. In total, 162 offspring born to mothers with PCOS (*n* = 48) and without PCOS (*n* = 114) were included. Forty-nine offspring were born prior to, and 113 after obesity surgery.

**Results:**

Mean ± SD birthweight (BW), birth length (BL), and head circumference (HC) before and after surgery for offspring born to mothers with PCOS were 3987 ± 495 g vs 3396 ± 526 g (*P* = 0.001), 52.2 ± 1.6 cm vs 50.1 ± 2.2 cm (*P* = 0.010), and 36.3 ± 1.97 cm vs 35.3 ± 1.66 cm (*P* = 0.183), respectively. In the non-PCOS group BW, BL and HC before and after were 3859 ± 603 g vs 3490 ± 538 g (*P* = 0.001), 51.3 ± 2.0 cm vs 49.9 ± 2.5 cm (*P* = 0.013), and 36.4 ± 2.0 cm vs 35.3 ± 1.8 cm (*P* = 0.016), respectively. Post-surgery, we found no difference in z-score BW, (∆–0.08, *P* = 0.677), BL (∆0.21, P = 0.184), and HC (∆0.14, *P* = 0.476) between children of PCOS and non-PCOS mothers.

**Comclusion:**

Babies born after obesity surgery were smaller and shorter in both the PCOS and non-PCOS group. Post-surgery anthropometrics were similar in babies born to mothers with and without PCOS.

## What does this study add to the clinical work

**Table:** 

Obesity surgery may decrease the negative effect of PCOS on offspring birth anthropometrics. Babies born after obesity surgery were smaller and shorter in both the PCOS and non-PCOS group, and post -surgery anthropometrics were similar in babies born to mothers with and without PCOS	

## Introduction

Polycystic ovary syndrome (PCOS) as well as obesity are associated with impaired fertility, adverse pregnancy outcomes, and may have a negative impact on offspring birth anthropometrics [1, 2]. Among pregnant women in Norway, the prevalence of obesity with body mass index (BMI) > 30 kg/m^2^ was 16% in 2022 [3]. More than half of the women with PCOS have obesity, which worsens their ovulatory disorder and risk of perinatal complications [4, 5]. Women with PCOS and obesity are recommended to lose 5–10% of their body weight to improve metabolic disturbances and reproductive outcomes [6].

Obesity surgery can be considered a treatment option for women with PCOS and obesity, which can contribute to weight loss and better metabolic status [2, 7–9]. The anatomical changes after obesity surgery lead to reduced nutritional intake and absorption, and changes in gut hormones [10]. Deficiencies of iron, vitamin B12, and vitamin D are common side effects, and malnutrition induced by obesity surgery may result in fetal growth restriction [11, 12]. Pregnancies following obesity surgery seem to be associated with preterm delivery, but also a lower risk of large for gestational age (LGA) offspring, and macrosomia [13]. A recent study from Finland found a higher risk of small for gestational age (SGA) offspring after surgery [14]. However, the factors that contribute to adverse birth outcomes after obesity surgery are not fully understood [15]. The negative effects on pregnancy outcomes after obesity surgery may be related to reduced fetal nutrition and/or placental function [16].

Women with PCOS have an increased risk of pregnancy complications, such as gestational diabetes, preeclampsia, preterm delivery, and an increased risk of small for gestational age (SGA) offspring [17]. The complications may be related to the metabolic, endocrine, and immunologic disturbances following PCOS [18]. After obesity surgery, women with PCOS report decreased burden of symptoms, decreased number of comorbidities, and remission of PCOS is also reported [2, 19]. A recent publication found similar rate of pregnancy complications in women with and without PCOS after obesity surgery [20].

As both PCOS and obesity surgery may increase the risk of offspring growth restriction, the aim of our study was to investigate the impact of obesity surgery on newborn anthropometrics in women with PCOS diagnosed before bariatric surgery compared to women without PCOS. The results were compared to a reference population using gestational age- and sex-adjusted Z-scores.

## Materials and methods

The present study includes data from the Bariatric Surgery Observation Study (BAROBS), and the Metformin in pregnant PCOS women study (PregMet2), supplemented with data from patients’ medical records.

BAROBS is an observational multicenter study, aimed to explore the diverse long-term effects of obesity surgery [21].The study included 321 women under the age of 45 years whounderwent Roux-en-Y gastric bypass (RYGB) in 2003–2009 and met to a follow-up visit in 2018–2020. Indications for surgery were preoperative BMI > 40 kg/m^2^ or BMI > 35 kg/m^2^ with obesity-related comorbidities. After RYGB, 66 of the women gave birth to a total of 101 offspring, and 17 (26%) of the women had been diagnosed with PCOS prior to surgery.

PregMet2 is a double-blinded, randomized trial that investigated whether metformin prevents late miscarriage and preterm delivery in women with PCOS [22]. The women were recruited from healthcare centers in Norway, Sweden, and Iceland in 2012–2017. Eleven women had either RYGB or sleeve gastrectomy, prior to study participation, five of them used metformin during the pregnancy but no differences in birth weight or birth length were found between metformin users and non-users [23]. None of the BAROBS participants used metformin during their pregnancies.

Women who did not give birth post-surgery, offspring born before gestational week 24, and offspring lacking data on pregnancy and birth characteristics in the regional electronic medical records were excluded from the analyses.

Some women gave birth to several offspring, and hence the study has been carried out with the offspring as the study participant. As each pregnancy is different with distinct maternal characteristics, each pregnancy has been counted as a separate event.

Maternal weight and BMI, before and after surgery, were retrieved from the BAROBS and PregMet2 databases. NADIR-weight was the lowest documented weight during the first 2 years after surgery. Pregnancy complications and mode of delivery, as well as data on offspring anthropometrics and gestational age (GA) were collected from the medical records.

Z-scores of newborn anthropometrics are standard deviations adjusted to sex and gestational age. z-scores express the deviation between the standard population and the observed values, and is regarded as a more accurate way of measuring and comparing growth and nutritional status [24]. Sex- and gestational age-adjusted z-scores for BW, BL, and HC were calculated from a reference population from six different industrialized countries [25].

Offspring with weight between the 10th and the 90th percentile were categorized as appropriate for gestational age (AGA), offspring below the 10th percentile as small for gestational age (SGA), and offspring above the 90th percentile as large for gestational age (LGA) [25]. Offspring of mothers with PCOS are categorized as the PCOS group, and offspring of mothers without PCOS are categorized as the non-PCOS group.

### Statistical analysis

Normally distributed continuous variables were reported as mean ± standard deviation (SD). Independent *t* test was used for comparison of normally distributed data, and Mann–Whitney *U* test elsewise. Categorical variables were reported as frequencies and percentages, and compared using Chi-square test, and Fisher Freeman Halton exact test. A *P* value less than 0.05 was considered as statistically significant as for all analyses. IBM SPSS Statistics, software version 28.01 was used for the statistical analyses.

## Results

**Fig 1:**
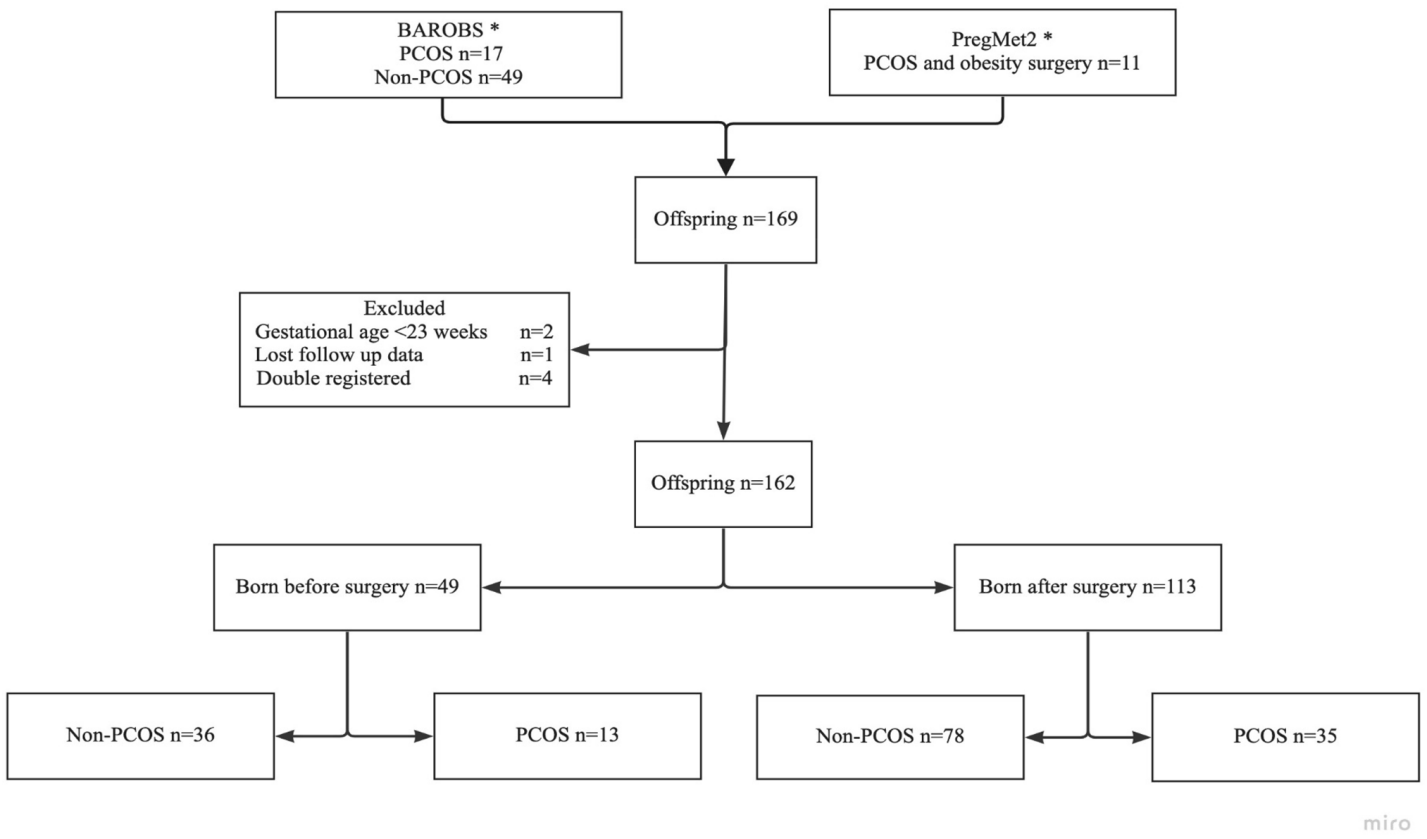
Flow chart of the included women and offspring. *Following the exclusion criteria, the total number of women were reduced from 77 to 72.

Seventy-two women gave birth to a total of 162 offspring, and 48 (63%) of them gave birth to more than one offspring. Thirty-two (44%) women gave birth both before and after obesity surgery, while 40 (56%) women gave birth only after surgery. A total of fourty-eight (30%) offspring were born to mothers with PCOS (Fig.1).

Sociodemographic data for mothers are shown in Table1. Maternal age (mean ± SD) at time of birth was 22.9 ± 3.9 years before surgery and 33.4 ± 4.8 years after. There were no major differences in socioeconomic status between the PCOS and non-PCOS groups. Weight and BMI were similar before and after surgery, and prior to pregnancy, in the two groups (Table2).

**Table 1:** Characteristics of mothers before and after obesity surgery, with and without PCOS

	Total	Pre-surgery			Post-surgery		
	*N* = 162	Non-PCOS *N* = 36	PCOS *N* = 13	*P* value	Non-PCOS *N* = 78	PCOS *N* = 35	*P* value
Age (years)	30.2 ± 6.7	22.8 ± 3.5	23.2 ± 5.1	0.724	33.4 ± 4.8	33.4 ± 5.1	0.948
Height (cm)	167 ± 6.6	166 ± 8.0	168 ± 5.1	0.394	168 ± 6.6	168 ± 5.7	0.756
Pre pregnancy weight (kg) (*n* = 125)	92.2 ± 18.9	101.6 ± 14.6	95.5 ± 13.8	0.488	91.4 ± 19.2	90.9 ± 16.6	0.899
Pre pregnancy BMI (kg/m^2^)	32.6 ± 6.2	36.1 ± 5.0	33.7 ± 5.0	0.426	32.2 ± 5.7	32.5 ± 7.4	0.849
< 30	41 (25.3)	2 (5.6)	1 (7.7)		25 (32.1)	13 (37.2)	
≥ 30	84 (51.9)	8 (27.8)	3 (30.8)		51 (65.4)	22 (62.9)	
Missing*	37 (22.8)	26 (72.2)	9 (69.2)		2 (2.6)	0	
Weight gain in pregnancy (kg) (*n* = 125)	10.9 ± 8.0	16.0 ± 9.3	16.0 ± 2.8	1.00	10.0 ± 7.8	11.1 ± 8.1	0.511
Educational level							
≤ 12 years	103 (63.6)	23 (63.9)	8 (61.5)	0.949	56 (71.8)	16 (45.7)	0.026^b^
> 12 years	53 (32.7)	12 (33.3)	3 (23.1)		21 (26.9)	17 (48.6)	
Missing	6 (3.7)	1 (2.8)	2 (15.4)		1 (1.3)	2 (5.7)	
Marital status				0.002^b^			0.735
Single	16 (9.9)	1 (2.8)	2 (15.4)		8 (10.3)	5 (14.3)	
Cohabitant	84 (51.9)	17 (47.2)	9 (69.2)		39 (50.0)	19 (54.3)	
Married	57 (35.2)	18 (50.0)	0		29 (37.2)	10 (28.6)	
Missing	5 (3.1)	0	2 (15.4)		2 (2.6)	1 (2.9)	
Smoking				0.293			0.901
Yes	48 (29.6)	13 (36.1)	2 (15.4)		22 (28.2)	11 (31.4)	
No	114 (70.4)	23 (63.9)	11 (84.6)		56 (71.8)	24 (68.6)	

Data are reported as mean ± SD or frequency, n (%). P values were non-significant. *BMI* body mass index, *LMP* last menstrual period. As the offspring are the participants, the same woman may have been counted several times, as each offspring have their respective mother. None of the mothers had T2DM during pregnancies, but three had T2DM before bariatric surgery and were still in remission. The frequency of other comorbidities prior to bariatric surgery was < 5%. *Pre pregnancy weight was missing in 37 cases; therefore, *n* = 125 for pre-pregnancy weight and BMI, as well as weight gain in pregnancy

[b] Fisher-Freeman-Halton Exact Test, significance *p*<0.05

**Table. 2:** Characteristics of mothers of offspring born after obesity surgery

Variables	Total *N* = 113	Non-PCOS *N* = 78	PCOS *N* = 35	*P* value
Pre-surgery weight (kg)	131 ± 17.2	131 ± 17.5	129 ± 16.4	0.507
Pre-surgery BMI (kg/m^2^)	46.3 ± 5.2	46.2 ± 4.8	46.1 ± 6.2	0.887
Post-surgery weight (kg)	104 ± 25.5	105 ± 5.9	103 ± 2.7	0.842
Post-surgery BMI (kg/m^2^)	36.7 ± 8.5	36.4 ± 0.8	37.7 ± 2.3	0.584
Post-surgery BMI, (kg/m^2^)				0.789
< 30	33 (29.2)	19 (24.4)	14 (40.0)	
≥ 30	80 (70.8)	59 (75.6)	21 (60.0)	
Weight reduction post-surgery* (kg)	27.1 ± 18.5	28.0 ± 17.0	29.2 ± 22.0	0.767
BMI reduction after surgery (kg/m^2^)	9.5 ± 6.7	9.8 ± 6.3	10.4 ± 7.8	0.728
Surgery to early pregnancy total weight loss (kg)	39.2 ± 14.3	40.0 ± 14.5	36.7 ± 13.5	0.304
Surgery to LMP time interval (months)	45.9 ± 33.8	42.4 ± 5.6	42.4 ± 5.6	0.493
< 24	35 (30.9)	24 (30.8)	11 (31.4)	
> 24	76 (67.3)	52 (66.7)	24 (68.6)	
Missing	2 (1.8)	2 (2.6)	0	

Data are reported as mean ± SD or frequency, n (%). P values were non-significant. *BMI* body mass index, *LMP* last menstrual period. *Lowest documented weight during the first two years after surgery. As the offspring are the participants, the same woman may have been counted several times, as each offspring have their respective mother

Neonatal anthropometrics and gestational age for offspring born to mothers with and without PCOS are presented in detail in Table3.

**Table 3:** Neonatal anthropometrics and gestational age, of offspring born to mothers with and without PCOS

	Total	PCOS			Non- PCOS		
Variables	*N* = 162	Pre-surgery *N* = 13	Post-surgery *N* = 35	*P* value	Pre-surgery *N* = 36	Post-surgery *N* = 78	*P* value
Sex, n (%)				0.668			0.521
Male	95 (58.6)	9 (69.2)	22 (57.1)		18 (50.0)	48 (61.5)	
Female	65 (40.1)	4 (30.8)	15 (42.9)		16 (44.4)	30 (38.5)	
Missing	2 (1.2)	0	0		2 (5.6)	0	
Birthweight							
Mean ± SD (g)	3592 ± 580	3987 ± 495	3396 ± 526	0.001^a^	3859 ± 603	3490 ± 538	0.001^a^
Z-score^*^ ± SD	0.15 ± 0.96	0.49 ± 1.10	−0.05 ± 0.78	0.068	0.56 ± 0.99	0.03 ± 0.95	0.013^a^
Percentile ± SD	54.0 ± 27.6	63.4 ± 30.7	47.3 ± 25.5	0.071	64.8 ± 26.4	51.3 ± 27.5	0.023^a^
Missing, *n* (%)	0	0	0		0	0	
Birth length							
Mean ± SD (cm)	50.4 ± 2.4	52.2 ± 1.6	50.1 ± 2.2	0.010^a^	51.3 ± 2.0	49.9 ± 2.5	0.013^a^
* Z*-score^*^ ± SD	−0.17 ± 0.79	0.13 ± 0.91	−0.13 ± 0.67	0.315	0.15 ± 0.78	−0.34 ± 0.79	0.007^a^
Percentile ± SD	45.1 ± 25.1	51.9 ± 27.9	46.0 ± 23.3	0.505	55.1 ± 25.9	40.3 ± 24.5	0.010^a^
Missing, n (%)	12 (7.4)	3 (23.1)	0		7 (19.4)	2 (2.6)	
Head circumference							
Mean ± SD (cm)	35.5 ± 1.8	36.3 ± 2.0	35.3 ± 1.7	0.183	36.4 ± 2.0	35.3 ± 1.8	0.009^a^
* Z*-score^*^ ± SD	0.50 ± 1.10	0.38 ± 1.50	0.47 ± 0.98	0.851	1.14 ± 1.40	0.33 ± 0.94	0.002^a^
Percentile ± SD	63.5 ± 28.6	58.8 ± 38.3	63.5 ± 26.4	0.709	76.6 ± 29.9	59.9 ± 27.7	0.015^a^
Missing, *n* (%)	21 (13.0)	7 (53.8)	0		12 (33.3)	2 (2.6)	
Birthweight < 2500 g, n (%)	8 (4.9)	0	1 (2.9)		1 (2.8)	6 (7.7)	
Birthweight > 4000 g, n (%)	34 (21.0)	5 (38.5)	5 (14.3)	0.108	12 (33.3)	12 (15.4)	0.053
Percentile groups, n (%)				0.443			0.261
SGA	12 (7.4)	1 (7.7)	2 (5.7)		2 (5.6)	7 (9.0)	
AGA	122 (75.3)	9 (69.2)	30 (85.7)		20 (55.6)	63 (80.8)	
LGA	19 (11.7)	3 (23.1)	3 (8.6)		6 (16.7)	7 (9.0)	
Missing	9 (5.6)	0	0		8 (22.2)	1 (1.3)	
Percentile groups birth length, *n* (%)				0.397			0.004^c^
≤ 10 percentiles	15 (9.3)	0	2 (5.7)		0	13 (16.7)	
≥ 90 percentiles	4 (2.5)	1 (7.7)	0		3 (8.3)	0	
Percentile groups head circumference, *n* (%)				0.189			0.003^b^
≤ 10 percentiles	6 (3.7)	1 (7.7)	7 (20.6)		1 (2.8)	3 (3.8)	
≥ 90 percentiles	35 (21.6)	2 (15.4)	1 (2.9)		12 (33.3)	13 (16.7)	
Gestational age							
Mean ± SD (weeks)	39.5 ± 1.7	40.8 ± 1.2	39.1 ± 1.7	0.007	39.8 ± 1.7	39.4 ± 1.8	0.318
Gestational age groups, *n* (%)				0.011^c^			0.576
Preterm < 37 weeks	8 (4.9)	0	2 (5.7)		1 (2.8)	5 (6.4)	
Term 37– 41 weeks	129 (79.6)	7 (53.9)	32 (91.4)	0.034^b^	22 (61.1)	68 (87.1)	0.734
Post-term > 42 weeks	11 (6.8)	3 (23.1)	1 (2.9)		3 (8.3)	4 (5.1)	
Missing	14 (8.6)	3 (23.1)	0		10 (27.8)	1 (1.3)	

*SD* standard deviation, *SGA* small for gestational age, *AGA* appropriate for gestational age, *LGA* large for gestational age. *BW* birthweight *Z-score is birthweight, birth length, head circumference adjusted for gestational age. ^a^ *t* test significance p < 0.05, ^b^ Mann–Whitney U, significance p < 0.05, ^c^ Fisher–Freeman–Halton exact test, significance *P* < 0.05

### Birthweight

For the entire population, BW and BW z-score (mean ± SD) were 3893 ± 574 g and 0.54 ± 1.0 before surgery, compared to 3460 ± 534 g and 0.01 ± 0.9 after (*P* < 0.001 and *P* = 0.002). In the PCOS group, BW was 3987 ± 495 g and z-score 0.49 ± 1.10 before surgery, compared to 3396 ± 526 g and z-score -0.05 ± 0.78 after *(P* = 0.001 and *P* = 0.068). In the non-PCOS group, BW was 3859 ± 603 g with z-score 0.56 ± 0.99 before surgery, and 3490 ± 538 g with z-score 0.03 ± 0.95 after (*P* = 0.001 and *P* = 0.013). There was no difference between the PCOS group and non-PCOS group, before or after surgery (Table4).

**Table 4:** Neonatal anthropometrics and gestational age, of offspring before and after obesity surgery

	Total	Pre-surgery			Post-surgery		
Variables	*N* = 162	PCOS *N* = 13	Non-PCOS *N* = 36	*P* value	PCOS *N* = 35	Non-PCOS *N* = 78	*P* value
Birthweight							
Mean ± SD (g)	3592 ± 580	3987 ± 495	3859 ± 603	0.497	3396 ± 526	3490 ± 538	0.385
* Z*-score^*^ ± SD	0.15 ± 0.96	0.49 ± 1.10	0.56 ± 0.99	0.853	–0.05 ± 0.78	0.03 ± 0.95	0.667
Percentile ± SD	54.0 ± 27.6	63.4 ± 30.7	64.8 ± 26.4	0.882	47.3 ± 25.5	51.3 ± 27.5	0.458
Missing, *n* (%)	0	0	0		0	0	
Birth length							
Mean ± SD (cm)	50.4 ± 2.4	52.2 ± 1.6	51.3 ± 2.0	0.216	50.1 ± 2.2	49.9 ± 2.5	0.660
* Z*-score^*^ ± SD	−0.17 ± 0.79	0.13 ± 0.91	0.15 ± 0.78	0.948	–0.13 ± 0.67	–0.34 ± 0.79	0.184
Percentile ± SD	45.1 ± 25.1	51.9 ± 27.9	55.1 ± 25.9	0.746	46.0 ± 23.3	40.3 ± 24.5	0.252
Missing, n (%)	12 (7.4)	3 (23.1)	7 (19.4)		0	2 (2.6)	
Head circumference							
Mean ± SD (cm)	35.5 ± 1.8	36.3 ± 2.0	36.4 ± 2.0	0.945	35.3 ± 1.7	35.3 ± 1.8	0.889
Z-score^*^ ± SD	0.50 ± 1.10	0.38 ± 1.50	1.14 ± 1.40	0.256	0.47 ± 0.98	0.33 ± 0.94	0.476
Percentile ± SD	63.5 ± 28.6	58.8 ± 38.3	76.6 ± 29.9	0.329	63.5 ± 26.4	59.9 ± 27.7	0.529
Missing, *n* (%)	21 (13.0)	7 (53.8)	12 (33.3)		0	2 (2.6)	
Percentile groups, *n* (%)							
SGA	12 (7.4)	1 (7.7)	2 (5.6)	0.951	2 (5.7)	7 (9.0)	0.558
AGA	122 (75.3)	9 (69.2)	20 (55.6)	0.889	30 (85.7)	63 (80.8)	0.634
LGA	19 (11.7)	3 (23.1)	6 (16.7)	0.908	3 (8.6)	7 (9.0)	0.945
Missing	9 (5.6)	0	8 (22.2)		0	1 (1.3)	
Gestational age							
Mean ± SD (weeks)	39.5 ± 1.7	40.8 ± 1.2	39.8 ± 1.7	0.069	39.1 ± 1.7	39.4 ± 1.8	0.283

*SD* standard deviation, *SGA* small for gestational age, *AGA* appropriate for gestational age, *LGA* large for gestational age. *BW* birthweight *Z-score is birthweight, birth length, head circumference adjusted for gestational age. ^a^ *t* test significance *P* < 0.05

The number of offspring with weight in the AGA category increased from 29 out of 41 (70%), to 93 out of 112 (83%) in the total population (*P* = 0.110), from 9 (69%) to 30 (86%) in the PCOS group (*P* = 0.230), and from 20 (56%) to 63 (81%) in the non-PCOS group (*P* = 0.280). Three (7%) offspring were classified as SGA before surgery, and nine (8%) offspring after (*P* = 1.00). Nine (22%) offspring were classified as LGA before surgery compared to ten (9%) offspring after (*P* = 0.049). There was no difference in AGA, LGA or SGA related to PCOS status (Table 3).

### Birth length

BL and BL z-score in the total population were 51.5 ± 1.9 cm and 0.14 ± 0.8 before obesity surgery compared to 50.0 ± 1.2 cm and −0.28 ± 0.8 after (*P* =  < 0.001 and *P* = 0.003). In the PCOS group, BL was higher before surgery compared to after surgery. Expressed in z-score, however, BL was similar before and after surgery. In the non-PCOS group, BL and BL z-score were higher before surgery compared to after surgery (Table 3). No difference between the PCOS and non-PCOS group was found when comparing BL and BL z-score, neither before nor after surgery.

### Head circumference

HC and HC z-score for the total population were lower after surgery, respectively, 36.4 ± 1.9 cm and 0.99 ± 1.4 before, and 35.3 ± 1.7 cm and 0.38 ± 1.0 after surgery (*P* = 0.003 and *P* = 0.019). There was no difference in HC or HC z-score in the PCOS group after surgery. However, HC and HC z-score were higher before than after surgery in the non-PCOS group. Offspring born to mothers with PCOS had a similar HC z-score as offspring born to mothers without PCOS before and after surgery (Table 3).

### Gestational age

In total, GA decreased from 40.1 ± 1.5 weeks prior to surgery, to 39.3 ± 1.7 weeks after (*P* = 0.023). GA was higher before than after in the PCOS group, while there was no difference in the non-PCOS group. When comparing GA in the two groups before and after surgery, no differences were found.

The rate of offspring born at term increased both in the PCOS and non-PCOS groups after surgery, from 7 (54%) to 32 (91%) (*P* = 0.034), and from 22 (61%) to 68 (85%) (*P* = 0.734), respectively. There was no difference between the two groups after surgery (*P* = 0.751) (Table 3).

## Discussion

The main findings in this study were that obesity surgery resulted in fetal growth restriction in both the PCOS and non-PCOS group. Offspring born to mothers with PCOS after obesity surgery were not more growth restricted than those born to mother without PCOS. Both anthropometric measurements and gestational age appeared to be normalized in offspring from mothers, both with and without PCOS, following obesity surgery, accompanied by a decrease in the incidence of LGA. No change was observed in SGA. Given the small number of PCOS pregnancies prior to obesity surgery, the results of this study should be interpreted with caution.

This study presents birth anthropometrics using z-scores, a method less frequently used in prior research. Most studies have reported these measurements using absolute values along with LGA and SGA. Using z-score, the results indicate more accurately the deviation from the normal population, and whether the offspring have a tendency of being SGA or LGA. We found that BW, BL, and HC z-score for the total population had a reduction and normalization following obesity surgery, in line with several earlier studies [12, 26].

According to former studies investigating the effect of PCOS and obesity surgery separately, we would assume that adding the impact of obesity surgery should give an additive growth restrictive effect on offspring born to mothers with PCOS [12, 13, 26]. The fact that we did not find this connection may be due to the limited number of PCOS pregnancies before surgery. A recent study found a lower BW in offspring born to mothers with PCOS compared to mothers without PCOS following obesity surgery, when BW was not adjusted for GA and sex [20]. The same study found no difference in SGA before and after obesity surgery, supporting our findings.

In addition to a reduction in absolute and z-score values for birth anthropometrics, there was a reduction in the total number of LGA offspring post-surgery in the total population, as reported in other studies [26, 27]. This is regarded as a positive effect of obesity surgery. However, an increase in the number of AGA offspring after surgery was not confirmed.

In our study, there was no increase in the number of SGA offspring after obesity surgery. These findings differ from most previous studies, which conclude that the risk of SGA offspring is an important negative consequence of obesity surgery [26, 28]. Since maternal malnutrition can be an important risk factor for SGA offspring after surgery, this result can reflect a more optimal follow-up of with micronutrient substitution during pregnancy in our population [12]. It has been shown that patients followed by a telephone nutritional management program had better perinatal outcomes, but the risk of SGA was not improved [29].

The strengths of this study are good quality data on birth anthropometrics, GA, and maternal data. Birth anthropometrics z-score was based on international standards of industrialized countries, which gives a precise estimate for difference in BW, BL, and HC, adjusted for GA and sex. The women included in this study were treated at public hospitals in Scandinavia, with a comprehensive post-operative follow-up and free of charge maternity care.

A limitation of the study is the sample size, in particular the low number of PCOS offspring before surgery, limiting the possibilities to reveal differences due to lack of statistical power.

In addition, there was some missing data on offspring born before obesity surgery. The data used in this study were mainly registered in a clinical setting over a long period of time, and the results should be interpreted with that in mind.

## Conclusion

The present study confirms that babies born after obesity surgery were smaller and shorter in both the PCOS and non-PCOS group. It also adds new knowledge about the impact of obesity surgery in women with PCOS, and their offspring. Post-surgery anthropometrics were similar in babies born to mothers with and without PCOS. Due to limited sample size, the results should be interpreted with caution.

## Data Availability

The authors confirm that the data supporting the findings of this study are available within the article. Raw data that support the findings of this study are available from the corresponding author, upon reasonable request.
